# Impact of the fear of Covid-19 infection on intent to breastfeed; a cross sectional survey of a perinatal population in Qatar

**DOI:** 10.1186/s12884-022-04446-z

**Published:** 2022-02-05

**Authors:** Shuja Mohd Reagu, Salwa Abuyaqoub, Isaac Babarinsa, Nisha Abdul Kader, Thomas Farrell, Stephen Lindow, Nahid M. Elhassan, Sami Ouanes, Noor Bawazir, Anum Adnan, Dina Hussain, Malika Boumedjane, Majid Alabdulla

**Affiliations:** 1 Mental Health Services, Hamad Medical Corporation, PO Box: 3050, Doha, Qatar; 2 Weill Cornell Medicine, Ar-Rayyan, Qatar; 3 Women’s Wellness Research Center, Hamad Medical Corporation, Doha, Qatar; 4 Coombe Women’s and Infants Hospital, Dublin, Ireland; 5 College of Medicine, Qatar University, Doha, Qatar

**Keywords:** Intent to breastfeed, Covid-19, Obsessive–Compulsive symptoms

## Abstract

**Objectives:**

Infection control measures during the Covid-19 pandemic have focused on limiting physical contact and decontamination by observing cleaning and hygiene rituals. Breastfeeding requires close physical contact and observance of hygienic measures like handwashing. Worries around contamination increase during the perinatal period and can be expressed as increase in obsessive compulsive symptoms. These symptoms have shown to impact breastfeeding rates. This study attempts to explore any relationship between the Covid-19 pandemic and perinatal obsessive–compulsive symptomatology and whether the Covid-19 pandemic has any impact on intent to breastfeed.

**Methods:**

A cross sectional survey of perinatal women attending largest maternity centre in Qatar was carried out during the months of October to December 2020. Socio-demographic information, intent to breastfeed and information around obsessive compulsive thoughts around Covid-19 pandemic were collected using validated tools.

**Results:**

15.7% respondents report intent to not breastfeed. 21.4% respondents reported obsessive–compulsive symptoms. 77.3% respondents believed the biggest source of infection was from others while as only 12% of the respondents believed that the source of infection was through breastfeeding and 15.7% believed the vertical transmission as the main source of risk of transmission.

**Conclusions:**

The rates of Obsessive–compulsive symptoms were increased and the rates of intent to breastfeed were decreased when compared with pre pandemic rates. The obsessive–compulsive symptoms and the intent to not breastfeed were significantly associated with fear of infection to the new-born.

Obsessive–compulsive symptoms were not significantly correlated with intent to breastfeed and can be seen as adaptive strategies utilized by women to continue breastfeeding in the context of fear of infection.

**Supplementary Information:**

The online version contains supplementary material available at 10.1186/s12884-022-04446-z.

## Background

Despite endorsement by major international healthcare and scientific bodies, the actual initiation of exclusive breastfeeding and its continuation still falls below targets worldwide and rates show widespread regional variation [1]. Rates in the Middle Eastern and North African region have been reported at 41% overall [2]. More recent studies looking at breastfeeding in Qatar in 2018, reported a high overall breastfeeding initiation rate of 96.2% but high discontinuation rates resulting in overall 6-month exclusive breastfeeding rates for of only 24.3% [3, 4].

There are emerging concerns that the breastfeeding initiation and continuation rates are being impacted, by the ongoing Covid-19 pandemic, through direct and indirect means. [5]. Despite the early recommendation from the WHO [6] around continuing breastfeeding, health services have been recently geared primarily towards pandemic control, and this has resulted in disruption of antenatal and postnatal services impacting the availability of breastfeeding support personnel and services [7, 8]. People are also limiting access to healthcare services due to fears around infection [9, 10].

Additionally, there are reported fears and misinformation around risks of vertical transmission of Covd-19 via breastmilk which however are not substantiated by evidence. [11, 12]. Systematic reviews and case reports of emerging evidence shows a low possibility of vertical transmission of the SARS- CoV-2 infection [12–15]. In fact, previous immunological studies on other respiratory infections like influenzas have indicated that transplacental antibodies protect the infant during the first few months of life from infection [16, 17]. However, this pandemic has been unprecedented not just in its spread but also the degree of misinformation surrounding it which has impacted traditionally held knowledge around communicable infections [18]. So, it is conceivable that concerns among mothers around transmission of the infection to their new-borns may potentially have a significant impact on breastfeeding. Indeed, a recent study in Qatar [19] highlighted concerns for their baby’s infection risks as one of the major pandemic-related worries among perinatal women.

The fundamental underpinning strategy for the control of the pandemic has been an emphasis on the prevention of spread of infection by avoiding close contact and ritualistic hand hygiene measures [20]. While a necessary measure, it has resulted in an increase in fears of contamination in the general public and vulnerable populations triggering obsessive compulsive type symptoms fuelled by fears of contamination [21, 22]. Breastfeeding is an act requiring close physical contact, and therefore can potentially be affected by these anxieties. It is known that pregnancy and delivery itself can precipitate obsessive compulsive symptoms around contamination which can negatively affect breastfeeding rates [23]. One of the known etiologic factors for the development of obsessive–compulsive disorder (OCD) symptoms is excessive fear and stress [24]; and studies have highlighted high levels of stress and anxiety among populations in the Covid-19 pandemic context, specifically among perinatal women [19, 25].

Against this background, it is important to explore the potential impact of Covid-19 related anxieties, fears of contamination and ritualistic infection control precautions on breastfeeding practices in order to inform public health interventions. It is within these contexts that this study attempts to explore any relationship between the impact of the Covid-19 pandemic on perinatal obsessive–compulsive symptomatology. It also explores whether the Covid-19 pandemic impacts breastfeeding rates through these symptoms.

## Methods

### Research design

A cross-sectional study design was chosen as this was an exploratory prevalence study in a clinical sample of pregnant women, in their third trimester, during the Covid-19 pandemic. Formal ethics approval for this study was obtained from the organizational Ethics Committee of Hamad Medical Corporation, Qatar where this study was undertaken (MRC-05–108). An information sheet detailing the project aims and scope was given to participants and informed written consent for participation obtained.

### Setting and relevant context

The state run, tertiary care maternity hospital in Qatar, where this study was conducted, is the largest provider of maternity services for the country and has a delivery rate of approximately 17,000 deliveries per year (unpublished data). Qatar has a unique demographic makeup with around 90% of its 2.7 million residents being economic migrants derived from all over the world [26]. As such native Qataris constitute roughly 10% of the population. However, since a significant number of economic migrants are manual and craft workers who do not come with their families, the proportion of Qatari females to non-Qatari females is higher than their male counterparts [26]. The hospital at which the study was conducted caters to all major population groups. Qatar is one of the wealthiest countries in the world experiencing rapid developments in infrastructure and education. The rates of initiation and continuation of breastfeeding have shown differences between local and immigrant population groups [3, 4]. For instance, the rate of continued breastfeeding upto one year has been reported as 4% among local Qatari women and 69.6% in immigrant women in Qatar. (5).

### Sample

Women in their third trimester attending the antenatal clinic or the inpatient services at largest state maternity hospital were approached for participation in this survey. Exclusion criteria included inability to consent or to engage with the written survey format due to literacy or learning difficulty issues or severe physical morbidity.

We calculated the sample size required considering both the intent not to breastfeed and the estimated prevalence of OCD symptoms in perinatal women. For the reported intent not to breastfeed in the same population at 3.8% [3], a desired precision at 4% and level of confidence limits at 95%, the required sample size for the 17,000 deliveries per year was determined at *n* = 88. Additionally, with an estimated prevalence of above 9% for OCD symptoms in perinatal women [27], a precision of 4%, and a level of confidence limits of 95%, the required sample size was determined at 196. To account for any possible refusals/withdrawals from the study, we inflated the sample size by one third, resulting in a sample size of 261. The participation was voluntary, with written consent and no payments or compensations were made to the respondents.

### Measurement

The survey questionnaire was a composite of two parts, where the first section collected relevant demographic data, information about the current pregnancy, past mental health history, self-reported concerns in relation to the pandemic, past breastfeeding experiences, intent to breastfeed in current pregnancy and coping factors perceived useful. Initial draft of the first section of the data collection tool was piloted on 20 patients by four researchers to assess feasibility of the data collection tool. Modifications were made to account for missing or unclear information and a final version was approved after discussion with the wider team. To achieve maximum reliability among the raters, two training sessions about the rating methods and terms were carried out.

The second part of the questionnaire was the validated instrument, the Yale-Brown Obsessive–Compulsive Scale-YBOCS symptoms scoring tool. The YBOCS is a standardized rating scale with both clinician-administered and self-report versions available, measuring 10 items pertaining to obsessions and compulsions on a five-point Likert scale. Scores range from 0 (no symptoms) to 4 (severe symptoms), and a total score is calculated by summing items 1 to 10 and can range from 0 to 40. It is a scale with demonstrated reliability and validity in measuring the severity of obsessive–compulsive symptoms and is not influenced by the type of obsessions or compulsions present [28, 29]. The composite questionnaire was translated into Arabic utilizing the translation and validation methodology described by Sousa & Rojjanasrirat [30]. The composite questionnaire was offered in both English and Arabic, the two main languages used in Qatar and the respondents were asked to respond to the YBOCS questions keeping in mind the context of the impact of the COVID 19 pandemic on them.

A score of eight and above on the YBOCS scale is indicative of mild Obsessive–Compulsive symptoms, more than fifteen indicative of moderate and more than 24 as indicative of severe Obsessive and Compulsive symptoms.

### Data collection

The survey was conducted in the months of October through to December 2020, when the COVID-19 pandemic in Qatar had passed its first peak and there was an ease in some of the restrictions of social distancing. Participation was offered to all pregnant women in their third trimester attending the hospital at the antenatal clinics, obstetric emergency unit and the in-patient maternity unit. Written consent was obtained from the respondents and the questionnaires were administered by clinicians. In total, 300 women were approached, out of whom 261 consented to participate in this study giving a response rate of 87%.

Data was collected on paper forms and no personal identifiers were recorded. Data forms were stored in locked lockers at the hospital and were entered electronically on secure institutional IT Systems protected by passwords.

Data collected were transferred to SPSS version 23.0 [31] for analysis. For descriptive statistics, we calculated the absolute and relative frequencies for categorical variables, and the mean and standard deviation for continuous variables.

To examine the associations between the intention to breastfeed and categorical variables, we used Pearson’s Chi-square test. In case of non-validity, we used Fisher’s exact test. To examine the associations between the intention to breastfeed and continuous variables, we the used the t test for independent samples.

The significance level was set at α = 0.05, and all tests were two-tailed.

## Results

The 261 respondents who completed the questionnaire had a mean age of 31 ± 5.5 years and a mean age of gestation at the time of filling the questionnaire of around 38 ± 2 weeks. Around one fifth (20.3%, *n* = 53) of the respondents were Qatari, and more than one third were non-Qatari Arab (37.3%, *n* = 97). In terms of level of education, most (59.3%, *n* = 155) were graduate education and 10% with higher degrees. Most respondents (83.3%, *n* = 220) had the intention to breastfeed their newborn (Table 1). Around one fifth of the respondents (21.4%, *n* = 56) scored over 8 on the Y-BOCS indicating mild OCD symptoms. As many as 5.7% (*n* = 15) of the respondents scored over 16 which indicated moderately severe OCD symptoms. One woman scored 25 which indicated severe OCD symptoms. This individual had an established diagnosis of OCD.

**Table 1:** General characteristics of the sample

**Age,** in years, m ± SD		31.0 ± 5.5
**Gestational age(in weeks),** m ± SD		38.3 ± 1.9
**Number of children,** m ± SD		2.3 ± 1.5
**Nationality,** n(%)	Qatari	53(20.3%)
	Non-Qatari Arab	97(37.3%)
	Indian Subcontinent	58(22.2%)
	Philippines	33(12.6%)
	Other	20(7.7%)
**Education level,** n(%)	Illiterate	1(0.4%)
	Primary	7(2.8%)
	Secondary	68(26.8%)
	Bachelor Degree or Equivalent	152(59.8%)
	Masters' Degree or Equivalent	22(8.7%)
	PhD Degree or Equivalent	4(1.6%)
**Mental health problems diagnosed in the past,** n(%)	No	252(97.3%)
	Depression	2(0.8%)
	Anxiety	2(0.8%)
	Psychosis	0(0%)
	Obsessive compulsive disorder	1(0.4%)
	Other	1(0.4%)
	Multiple	1(0.4%)
**Mental health treatment in the past,** n(%)		8(3.1%)
**Breastfed a child before,** n(%)		166(83.8%)
**Previous breast feeding problems,** n(%)		29(23.4%)
**Intention to breast feed,** n(%)		220(83.3%)
**Y-BOCS total score,** m ± SD		3.4 ± 5.3

*m* mean, * SD* standard deviation, *Y-BOCS* Yale-Brown Obsessive Compulsive Scale

  None of the sociodemographic or clinical factors (including age, gestational age, nationality, level of education, previous breastfeeding, previous problems with breastfeeding, history of mental health problems or treatment, and Y-BOCS score) was associated with the intention to breastfeed (Table 2).

**Table 2:** Associations between the intention to breastfeed and the general characteristics

	**Intention to breast feed** **(*n* = 220)**	**Do not intend to breast feed** **(*n* = 41)**	***p* value**
**Age,** in years, m ± SD	31.1 ± 5.4	30.4 ± 5.8	0.386
**Gestational age,** in years, m ± SD	38.5 ± 2.0	38.3 ± 3.2	0.242
**Nationality, n(%)**			
Qatari	41(18.6%)	12(29.3%)	0.504
Non-Qatari Arab	86(39.1%)	11(26.8%)	
Indian Subcontinent	47(21.4%)	11(26.8%)	
Philippines	29(13.2%)	4(9.8%)	
Other	17(7.7%)	3(7.3%)	
**Education**			
University level	149(69.6%)	29(72.5%)	0.437
Below university level	65(30.4%)	11(27.5%)	
**Previously breast fed a baby**			
Yes	137(85.6%)	29(76.3%)	0.125
No	23(14.4%)	9(23.7%)	
**Previous breast-feeding problems**			
Yes	24(24.7%)	5(18.5%)	0.346
No	73(75.3%)	22(81.5%)	
**History of mental health problems or treatment**			
Yes	5(2.3%)	3(7%)	0.126
No	215(97.7%)	40(93%)	
**Y-BOCS score,** m ± SD	3.3 ± 5.5	3.9 ± 5.0	0.521

*m* mean, * SD* standard deviation, *Y-BOCS* Yale-Brown Obsessive Compulsive Scale

Table 3 shows the most notable concerns that the pregnant women had in relation to the Covid-19 pandemic during the pregnancy and their associations with intent to breastfeed. The biggest worry by far was the impact of Covid-19 on their children and family (*n* = 164, 63%). Majority of the women *n* = 202 (77%) in this study stated that their biggest worry was that their baby might catch the infection. The concerns around the impact on pregnancy and breastfeeding were noted as second most frequent. None of the aforementioned concerns was found to be associated with the reported intention to breastfeed. Nearly half (47.5%) of the pregnant women in this study found self-sought information from Social media and TV the most helpful in managing concerns around Covid-19 followed by support from their friends and family. We found that women who relied on information from TV/media were less likely to have the intention to breastfeed (Table 3). Only *n* = 31(12%) of the women in this study believed that their children might catch the infection through breastfeeding or close intimate contact, and *n* = 41 (15.7%) women believed that there was a risk of vertical transmission. The biggest worry by far for majority of the women (*n* = 202, 77.3%), was that their child might catch the infection from family or friends or other unknown sources. Women who perceived that the main source for infection for their newborns was family and friends were more likely to have the intention to breastfeed (Table 3).

**Table 3:** Associations between the intention to breastfeed and COVID-19 worries, source of information, and infection transmission

	**Total** **(*n* = 261)**	**Intention to breast feed** **(*n* = 220)**	**Do not intend to breast feed (*n* = 41)**	***p* value**
**Covid-19: most impact on**				
Pregnancy	104(39.8%)	86(39.1%)	18(43.9%)	0.507
Breast feeding	32(12.3%)	28(12.7%)	4(9.8%)	0.811
Finances/job	35(13.4%)	32(14.5%)	3(7.3%)	0.225
Family and children	164(62.8%)	144(65.5%)	20(48.8%)	0.085
Other	10(3.8%)	9(4.1%)	1(2.4%)	0.481
**Sources of help in this pregnancy**				
Information from maternity staff	73(28.0%)	64(29.1%)	9(22.0%)	0.350
Information from TV / media	124(47.5%)	97(44.1%)	27(65.9%)	0.010
Family / friends	92(35.3%)	77(35.0%)	15(36.6%)	0.845
Hobbies	15(5.8%)	14(6.4%)	1(2.4%)	0.321
Exercise	21(8.1%)	19(8.6%)	2(4.9%)	0.417
Other	12(4.6%)	8(3.6%)	4(9.8%)	0.086
**Do you have concerns about transmission of infection to your new-born?**				
Yes	201(89.7%)	186(89.4%)	15(93.8%)	0.582
No	23(10.3%)	22(10.6%)	1(6.3%)	
**What would be the likely source of infection to your baby?**				
Close contact e.g. breast feeding	31(11.9%)	29(13.2%)	2(4.9%)	0.131
Family and friends	124(47.5%)	111(50.5%)	13(31.7%)	0.027
Pregnancy and delivery	41(15.7%)	34(15.5%)	7(17.1%)	0.794
Do not know	78(29.9%)	59(26.8%)	19(46.3%)	0.012

We also carried out multiple linear regression analysis and the analyses showed that the total Y-BOCS score was only positively associated with the reported COVID impact on breastfeeding (B = 4.328[0.398; 8.258], *p* = 0.031, *r* = 0.247) (Table 4).

**Table 4:** Variables associated with the Yale-Brown Obsessive Compulsive Scale total score—multiple linear regression analysis

**Variable**	**B**	**p**	**95% Confidence interval for B**		**Partial correlation coefficient**
			**Lower Bound**	**Upper Bound**	
**Age**	-.004	.983	-.343	.336	-.002
**Gestational age**	.296	.455	-.491	1.084	.087
**Educational level**	2.749	.061	-.129	5.627	.216
**Breastfed a child before**	-.646	.766	-4.963	3.671	-.035
**History of breastfeeding problems**	4.624	.230	-2.981	12.230	.139
**History of mental health problems/treatment**	-.038	.981	-3.281	3.205	-.003
**Breastfeeding intention**	1.315	.468	-2.274	4.903	.085
**Reported COVID impact on pregnancy**	-.077	.965	-3.579	3.424	-.005
**Reported COVID impact on breastfeeding**	**4.328**	**.031**	**.398**	**8.258**	**.247**
**Reported COVID impact on finances/job**	-2.636	.304	-7.710	2.439	-.119
**Reported COVID impact on family/children**	1.651	.350	-1.849	5.151	.109
**Reported COVID impact—Other**	-4.104	.242	-11.039	2.831	-.136
**Sources of help – Information from maternity staff**	1.216	.443	-1.926	4.359	.089
**Sources of help – Information from TV/Media**	-1.026	.468	-3.830	1.777	-.084
**Sources of help – Family/friends**	-.145	.924	-3.157	2.868	-.011
**Sources of help – Hobbies**	-1.575	.586	-7.306	4.157	-.064
**Sources of help – Exercise**	3.949	.101	-.784	8.683	.190
**Sources of help – Other**	-4.792	.372	-15.426	5.841	-.104
**Concerns about transmission of infection to newborn**	-.721	.714	-4.624	3.183	-.043

The binary logistic regression showed that only the number of children was positively associated with the intent to breastfeed (*p* = 0.022, OR = 37.437[1.675; 836.630]) (Table 5).

**Table 5:** Binary logistic regression showing the variables associated with the intent to breastfeed

	B	Wald	Sig	Exp(B) = OR	95% C.I.for EXP(B)	
					Lower	Upper
**Age**	-.204	.782	.377	.816	.519	1.281
**Children**	**3.623**	**5.223**	**.022**	**37.437**	**1.675**	**836.630**
**Qatari Citizen**	-2.875	1.758	.185	.056	.001	3.956
**Education level (university vs lower)**	-6.845	1.473	.225	.001	.000	67.272
**Pregnancy complications**	2.635	1.583	.208	13.937	.230	844.031
**Gestational age**	.161	.086	.769	1.174	.402	3.429
**Breastfed a child before**	-19.759	.000	.998	.000	.000	
**Previous mental health problems**	-23.237	.000	.998	.000	.000	
**Previous breastfeeding problems**	2.479	1.202	.273	11.928	.142	1001.812
**YBOCS total score**	.196	1.595	.207	1.217	.897	1.649
**Reported COVID impact on pregnancy**	3.838	2.427	.119	46.435	.371	5807.285
**Reported COVID impact on breastfeeding**	-4.809	1.750	.186	.008	.000	10.136
**Reported COVID impact on finances/job**	-.841	.083	.773	.431	.001	130.264
**Reported COVID impact on family/children**	3.407	2.729	.099	30.184	.530	1719.829
**Reported COVID impact—Other**	35.275	.000	.998	2,087,750,055,532,148	.000	
**Sources of help – Information from maternity staff**	2.153	.715	.398	8.609	.059	1264.173
**Sources of help – Information from TV/Media**	-2.805	1.810	.178	.061	.001	3.600
**Sources of help – Family/friends**	-.401	.084	.772	.670	.044	10.101
**Sources of help – Hobbies**	-.855	.097	.756	.425	.002	93.158
**Sources of help – Exercise**	-4.894	1.188	.276	.007	.000	49.645
**Sources of help – Other**	34.053	.000	.999	615,221,330,941,862	.000	
**Concerns about transmission of infection to newborn**	-.251	.009	.925	.778	.004	147.740
**Likely source of infection to new born (Reference = None)**		2.163	.706			
**Close contact e.g. breast feeding**	9.076	.000	1.000	8745.056	.000	
**Family and friends**	26.468	.000	.998	312,415,175,063.172	.000	
**Pregnancy and delivery**	2.780	1.935	.164	16.119	.321	809.906
**Do not know**	-1.022	.157	.692	.360	.002	56.522

## Discussion

To our knowledge, this is the first study of its kind in the MENA region that explores the impact of Covid-19 pandemic on intent to breastfeed. It is also the first study, as far as we are aware, that uses validated tools measuring OCD symptomatology to explore whether beliefs around fear of infection through close physical contact, like that in breastfeeding and undertaking of decontamination rituals impacts on breastfeeding intentions.

The two main findings of this study were that a high proportion of women at 15.7% expressed intent not to breast feed and more than 21% of respondents reported obsessive compulsive type symptoms around the Covid-19 pandemic. In comparison, the pre-pandemic percentages for these in the same population group are 3.8 and 9% respectively [3, 27]. Interestingly, however, this study found no direct correlation between the high scores on obsessive compulsive symptoms during the pandemic and the decrease in the intent to breastfeed. It is important to note that except for one individual, none of the participants had an established diagnosis of OCD and although a response to fears of contamination can lead to increased obsessive and compulsive rituals, those scoring above the cut offs are displaying concerns to the extent that are over and above the normal responses.

The only significant association was found to be between the fear of impact of Covid-19 pandemic on breastfeeding and Y-BOCS scores.

There were no associations between fears of infection and intent to breastfeed. When taken together with the finding that majority of the women believed that they themselves were unlikely to be the source of infection, a possible explanation begins to emerge. It can be argued based on these findings that these women see the obsessive thoughts about fear of infection and compulsive rituals to clean and prevent infection as a good enough strategy to prevent the infection to the new-born from others and from unknown sources. In fact, the high scores on obsessive–compulsive symptoms may represent the adaptive strategies adapted by lactating women to be able continue breastfeeding safely and reduce the risk of infection to the new-born in this overwhelming pandemic.

While the intention to breastfeed does not appear to be directly related to the obsessive–compulsive symptoms, it remains to be seen on how these may potentially impact continuation of breastfeeding, Continuation of breastfeeding requires ongoing engagement and effort and has traditionally been an area of poor performance within the MENA region and worldwide [2–5]. While women appear motivated to use ritual hygiene practices as a protective strategy while initiating breastfeeding, it has to be borne on mind that these have to be carried on over a prolonged period of several months. It is possible that this may be burdensome in the longer term for women who do start breastfeeding and therefore may result in early discontinuation. There is evidence that OCD sufferers show lower rates of breastfeeding [23].

This study was carried out at the time when the intensity of the Covid-19 pandemic was past its first peak in Qatar and there was some ease in restrictions and general public concern over the pandemic and its impact. Restrictions around social contacts had been relaxed [32]. Therefore, rates of intention to breastfeed may have been lower and anxieties around infection even higher at the peak of the epidemic, and indeed, may change again with successive peaks in infection rates with fresh waves of infection. The introduction of vaccination, its acceptance among breastfeeding mothers and its impact on intent to breastfeed is another important factor that will play an increasingly important role [33]. As this pandemic continues to unfold, presenting with new challenges, the need to integrate new research findings during Covid-19 into breastfeeding and other aspects of pregnancy and child birth, and emergency response planning for future emergencies has been highlighted as a public health priority [34].

By far the biggest worry for the women in this study around the Covid-19 pandemic was the impact of the pandemic on their children and family over and above concern for their own wellbeing. This is a finding that has been replicated in another study exploring the psychological impact of Covid-19 on perinatal women in Qatar [19]. This knowledge can serve to inform perinatal service planning, information campaigns as well public health measures in the current and future health crises.

Finally, majority of the women reported that they sought usable information around Covid-19 pandemic and safe breastfeeding from TV, social media or friends and family. This trend has been replicated in other studies exploring trusted sources of information among residents in Qatar during Covid-19 [35, 36]. This emerging pattern of reliance on self-research rather on traditional trusted sources of information like healthcare professionals is an important factor that can be utilised by healthcare policy makers in future. Healthcare policy makers need to adapt and utilise social media platforms to reach audiences with correct and up to date evidence and work closely with social media companies.

### Limitations

This study was conducted at the time when the Covid-19 pandemic was just past its first peak and represents a snapshot during an evolving health crisis. As the situation unfolds, rates of obsessive–compulsive symptoms and anxieties within the population may potentially change.

The sample was a convenience sample and although English and Arabic are widely spoken in Qatar, it excludes people not speaking these two languages.

## Conclusion

This study highlights that the Covid-19 pandemic has negatively impacted the intent to breastfeed among pregnant women in Qatar who constitute a vulnerable group. This impact appears to be attributable more to fear of infection to the new-born from close contacts rather than vertical infection or through breastfeeding prompting obsessive thoughts and rituals of decontamination among this group to be able to continue breastfeeding. Public health policy should be informed by the preference of women to seek information from social media and develop innovative strategies that educate about community transmission and hygienic practices for breastfeeding during this and any future pandemics.

It is important to further explore whether these obsessive–compulsive symptoms will further impact breastfeeding continuation rates which have traditionally been low in Qatar.

## Supplementary Information

**Additional file 1.**

## Data Availability

The data that support the findings of this study are available from the corresponding author upon reasonable request and pending additional ethical approval.

## Abbreviations

Covid-19: Coronavirus disease of 2019
SARS CoV 2: Severe Acute Respiratory Syndrome Coronavirus
WHO: World Health Organization
OCD: Obsessive Compulsive Disorder
YBOCS: Yale-Brown Obsessive–Compulsive Scale
MENA: Middle Eastern and North African
