# Supplementary material for: Impact of the fear of Covid-19 infection on intent to breastfeed; a cross sectional survey of a perinatal population in Qatar
Source: BMC Pregnancy Childbirth. 2022 Feb 5;22:104. doi: 10.1186/s12884-022-04446-z (PMC8817146; doi:10.1186/s12884-022-04446-z)
Supplement: Supplementary file 1 — Additional file 1. [file 12884_2022_4446_MOESM1_ESM.docx]

***For office use only***

| Type of encounter (OPC/In-pt) | Date | Number assigned |
| --- | --- | --- |
|  |  |  |

**Start of Questionnaire**

| Age | Number of children | Nationality | Educational level | Occupation |
| --- | --- | --- | --- | --- |
|  |  |  |  |  |

| How many weeks/months pregnant are you? | Do you have any medical complications in this or previous pregnancy (Diabetes/Blood pressure etc) |
| --- | --- |
|  |  |

| Have you ever sought help or received treatment for mental health problems? | Yes No |
| --- | --- |
| If Yes, what was the problem?  (Tick all that apply) | Depression  Anxiety  Psychosis  Fear of contamination and dirt/OCD  Other |
| If yes, what treatment or help did you receive?  (Tick all that apply) | Medication  Hospital admission  Counselling  Other |

| In relation to the COVID-19 pandemic, what are you most worried about?  (Choose one) | Effect on pregnancy  Effect on breastfeeding  Effect on Finances/Job  Effect on personal health  Effects on health of children and family  Other – please give details |
| --- | --- |

| What is your primary source of information for breastfeeding during this pregnancy?  (Choose one) | Information from the hospital maternity staff  Information from TV and social media  Information from friends and family  Other- please give details |
| --- | --- |

| Have you breastfed your previous child/children?  If Yes, how long did you continue for? | Yes  No  Not applicable  Exclusively: less than 1 month-  1 month to six months-  Mixed feeding of any duration: months |
| --- | --- |
|  |  |
| Did you face any problems with breastfeeding before? | Yes-  No-  Not applicable- |
| Do you intend to breastfeed after this delivery? | Yes-  No- |
| If you answered NO to the previous question, why?  (Tick all that apply) | Previous poor experience-  Fear of COVID-19 infection/contamination-  Do not choose to breastfeed-  Undecided-  Other- |
| What will you do differently this time during breastfeeeding? | Will not breastfeed-  Wash hands before and after each feed-  Wash breast before and after each feed-  No extra precautions- |
| Are you concerned about your child getting infected? | Yes-  No- |
| What is the most likely source of infection for your baby?  (Choose one) | From close contact with you during breastfeeding-  From family members/household contact-  During delivery or pregnancy-  Don’t know- |
